# Development and Evaluation of a Positive Youth Development Course for University Students in Hong Kong

**DOI:** 10.1100/2012/263731

**Published:** 2012-04-19

**Authors:** Daniel T. L. Shek, Rachel C. F. Sun, Y. H. Chui, S. W. Lit, Walter W. Yuen, Yida Y. H. Chung, S. W. Ngai

**Affiliations:** ^1^The Hong Kong Polytechnic University, Hong Kong; ^2^Faculty of Education, The University of Hong Kong, Hong Kong

## Abstract

With higher education, university graduates are important elements of the labor force in knowledge-based economies. With reference to the mental health and developmental problems in university students, there is a need to review university's role in nurturing holistic development of students. Based on the positive youth development approach, it is argued that promoting intrapersonal competencies is an important strategy to facilitate holistic development of young people in Hong Kong. In The Hong Kong Polytechnic University, a course entitled Tomorrow's Leader focusing on positive youth development constructs to promote student well-being will be offered on a compulsory basis starting from 2012/13 academic year under the new undergraduate curriculum structure. The proposed course was piloted in 2010/11 school year. Different evaluation strategies, including objective outcome evaluation, subjective outcome evaluation, process evaluation, and qualitative evaluation, are being carried out to evaluate the developed course. Preliminary evaluation findings based on the piloting experience in 2010/11 academic year are presented in this paper.

## 1. Introduction

There are research findings showing that adolescent developmental problems such as substance abuse, violence and mental health problems are intensifying in high school students [1]. In response to such adolescent developmental problems, there are adolescent prevention and positive youth development programs specifically designed for high school students. For example, a review of the programs in the Centre for Substance Abuse Prevention, Substance Abuse and Mental Health Service Administration Department of Health and Human Services, U.S. Government, showed that there are hundreds of programs developed for high school students and some of them have been shown to be effective in reducing adolescent risk behavior [2].

Comparatively speaking, there are fewer preventive programs for college students who usually transit to early adulthood from adolescence during their college years [3, 4]. Logically speaking, when high school students enter colleges, it is expected that developmental issues observed in high schools do not disappear overnight. As such, it is important to ask how university education could, would, and should help university students, who are normally in their late teens and early twenties, to thrive. Furthermore, it is important to ask how university education can help to promote holistic development of college students, particularly in the areas of responsible citizenship and engagement in civic responsibilities. With reference to Hong Kong, developmental issues and needs of college students in Hong Kong are reviewed in this paper. Adopting the approach of positive youth development, it is argued that offering credit-bearing courses to develop the psychosocial competencies of college students is a promising approach to promote holistic development in college students. Experiences based on the implementation of a course titled Tomorrow's Leaders at The Hong Kong Polytechnic University in 2010/11 school year are described and initial evaluation findings are presented.

There are several observations regarding adolescent development in Hong Kong [3]. First, research and statistics show that there are several developmental issues in young people in Hong Kong, such as growing adolescent substance abuse and worsening mental health of young people. For example, it is estimated that around 20% of young people in Hong Kong have psychosocial and adjustment problems in different domains [5]. Research findings also show that young people in Hong Kong are quite politically apathetic and they do not have a good understanding of the Hong Kong society as well as China [3].

Second, research findings suggest that poor mental health among university students is an issue deserving our attention. For example, there were high rates of emotional and anxiety problems in first year tertiary education students in Hong Kong. Third, findings based on employer surveys commonly showed that employers in Hong Kong were not satisfied with the personal qualities of graduates such as maturity, sense of responsibility, and communication. Fourth, although “whole person development” and “personal development” are commonly mentioned in the mission and vision statements of different universities in Hong Kong, attention is usually given to the intellectual development of university students (e.g., focus on Western and Chinese cultures) and universities have only paid lip service to holistic development of students.

One striking example reflecting the lack of nurturance of psychosocial competencies in university students can be seen in a case reported by the media in early 2011. The case is about a university graduate who had received undergraduate and postgraduate training and had good public and internal examination results. However, he failed to get a job after sending out more than 200 application letters and eventually applied for comprehensive social security allowance. When he was interviewed by the media, he stated that he might lack social competence but he claimed that such knowledge was not taught in the formal curriculum in school settings. From this case, the public asks one simple question: to what extent universities have nurtured university students in a holistic manner so that they can live and work well in early adulthood?

## 2. Holistic Youth Development in University Students

In the field of developmental psychology, one common criticism is that excessive focus has been put on adolescent problems. As such, an alternative approach emphasizing the importance of positive youth development has been proposed. Damon [6] stated that the field of positive youth development (PYD) focuses on each child's talents, strengths, interests, and future potential in contrast to approaches that focus on youth developmental problems such as delinquency and substance abuse. Catalano et al. [7] pointed out that there are several characteristics associated with the positive youth development approach, including focus on integrated youth development (i.e., attending to a wide range of youth developmental possibilities and problems) rather than dealing with a single youth problem, upholding the belief that “problem-free is not fully prepared”, emphasis of person-in-environment perspective, and focus on developmental models on how young people grow, learn, and change.

There are different models focusing on the importance of promoting adolescent developmental assets. For example, Benson [8] proposed 40 developmental assets for adolescents. With reference to such models, the goal of positive youth development programs is to cultivate adolescent developmental assets. Besides, there are many researchers suggesting that building cognitive, academic, social, and emotional competence is a fundamental task in adolescence. With reference to the specific assets to be developed, Weissberg and Utne O'Brien [9] proposed five core social-emotional competencies to be targeted in positive youth development programs: self-awareness, social awareness, self-management, relationship skills, and responsible actions.

Graczyk et al. [10] argued that the promotion of social and emotional learning (SEL) of adolescents can serve as a unifying framework that integrates both the risk and protective factors paradigm and the competence enhancement paradigm. According to the Collaborative for Academic, Social and Emotional Learning [11], “social and emotional learning (SEL) is the process of acquiring the skills to recognize and manage emotions, develop caring and concern for others, make responsible decisions, establish positive relationships, and handle challenging situations effectively. Research has shown that SEL is fundamental to children's social and emotional development—their health, ethical development, citizenship, academic learning, and motivation to achieve. Social and emotional education is a unifying concept for organizing and coordinating school-based programming that focuses on positive youth development, health promotion, prevention of problem behaviors, and student engagement in learning”. Generally speaking, several SEL attributes are commonly included in different SEL models. These include self-awareness (identifying emotions and recognizing strengths), social awareness (perspective-taking and appreciating diversity), self-management (managing emotions and goal setting), responsible decision making (analyzing situations, assuming personal responsibility, respecting others, problem solving), and relationship skills (communication, building relationships, negotiation, refusal). Sun and Shek [12] showed that higher positive youth development predicted lower problem behavior, thus suggesting that positive youth development is an important protective factor for adolescent problem behavior.

With specific reference to university education, Bok [13] argued that universities should help students develop the following attributes: ability to communicate, critical thinking, moral reasoning, preparation for citizenship, living with diversity, living in a global society, development of a breadth of interests, and preparation for work. According to the Council for the Advancement of Standards for Higher Education [14], the following developmental outcomes are important for nurturing the leadership qualities of university students: intellectual growth, effective communication, enhanced self-esteem, realistic self-appraisal, clarified values, career choices, leadership development, healthy behavior, meaningful interpersonal relationships, independence, collaboration, social responsibility, satisfying and productive lifestyles, appreciating diversity, spiritual awareness, and personal and educational goals.

Unfortunately, there are views suggesting that higher education in the contemporary world ignores holistic development of university students in reality. In a special issue on the inner lives of university students, Dalton and Crosby [15] pointed out that higher education pays very little attention to the inner lives of university education and that “educational and student development efforts that ignore students' spirituality, that is, how they make internal connections to the defining beliefs and purposes in their lives, will inevitably be less effective since they do not reach that part of students' lives where things really matter” (p. 1).

## 3. Tomorrow's Leaders: A Version of the Project P.A.T.H.S. in the University Context

In an age of accountability, educators are more concerned about student outcomes. Unfortunately, while outcomes in tertiary education are commonly related to academic and occupational domains, university administrators only pay lip service to holistic development in young people. As argued provocatively by Astin and Sax [16], “although we argued that institutions needed to focus more on student outcomes, we avoided specifying what any of these outcomes should be, arguing instead that this task should be left largely to the individual institution. In retrospect, I think this was a mistake. If we had been more forthcoming about our own values with respect to some of the most important student outcomes, we certainly would have generated more controversy, but I think the controversy would have been healthy. More specifically, I wish we had spoken more directly about the importance of so called affective outcomes such as self-understanding, tolerance, honesty, citizenship, and social responsibility” (p. 587). 

Against such a background and adopting the argument that adolescent developmental issues do not disappear over-night, Shek and Wong [4] argued that the development of credit-bearing courses on positive youth development would be helpful to nurture university students and proposed several principles in the development of such courses. These included holistic student development, responding to community concern about young people, preparing students for adulthood and general education with life-long benefits, uniqueness, universal coverage, theory-driven general education program, and research-driven general education program. As far as the model of positive development is concerned, Shek [3] argued that universities should help develop 14 positive youth developmental assets identified by Catalano et al. [7] based on effective positive youth development programs. The constructs are as follows:

1. promotion of bonding: development of relationships with healthy adults and positive peers in the extrafamilial and intrafamilial context;
2. cultivation of resilience: promotion of capacity for adapting to change and stressful events in healthy and adaptive ways;
3. promotion of social competence: promotion of interpersonal skills and providing opportunities to practice such skills;
4. promotion of emotional competence: development of skills to recognize feelings in oneself and others, skills to express feelings, skills to manage emotional reactions or impulses, and emotional self-management strategies;
5. promotion of cognitive competence: promotion of cognitive abilities, processes or outcomes, including academic performance, logical thinking, critical thinking, problem-solving, decision making, planning and goal setting, and self-talk;
6. promotion of behavioral competence: cultivation of verbal communication, non-verbal communication and taking action skills, and providing reinforcement for the effective behavior choices and action pattern;
7. promotion of moral competence: development of a sense of right and wrong, and respect for rules and standards as well as social justice;
8. cultivation of self-determination: promoting program participants' sense of autonomy, independent thinking, or self-advocacy;
9. promotion of spirituality: helping program participants to develop purpose and meaning in life, hope, or beliefs in a higher power;
10. development of self-efficacy: promoting coping and mastery skills and changing program participants' negative self-efficacy expectancies or self-defeating cognitions;
11. development of clear and positive identity: promotion of healthy identity formation and achievement, including positive identification with one's social or ethnic identity;
12. promotion of beliefs in the future: helping program participants to develop future potential goals, choices or options;
13. providing opportunities for prosocial involvement: designing activities and events for program participants to make positive contribution to groups;
14. fostering prosocial norms: encouraging program participants to develop clear and explicit standards for prosocial engagement.

Actually, these constructs are covered in the positive youth development framework in the Project P.A.T.H.S. in Hong Kong [17]. To promote holistic development among Hong Kong adolescents, The Hong Kong Jockey Club Charities Trust approved HK$400 million in 2005 to launch a project entitled P.A.T.H.S. to Adulthood: A Jockey Club Youth Enhancement Scheme. The word “P.A.T.H.S.” denotes Positive Adolescent Training through Holistic Social Programs in 2006/07 to 2008/09 school years. The Trust has invited academics of five universities in Hong Kong to form a Research Team with The Hong Kong Polytechnic University as the lead institution to develop a multiyear universal positive youth development program to promote holistic adolescent development in Hong Kong. Because of the positive impacts on the program participants, the Trust earmarked an additional grant of HK$350 million to support the project for another cycle from 2009/10 to 2011/12 school years. Different evaluation strategies have been employed to evaluate the program, including objective outcome evaluation, subjective outcome evaluation, interim evaluation, process evaluation, qualitative evaluation, and evaluation based on personal construct psychology. To date, evaluation findings strongly suggest that the Project P.A.T.H.S. is effective in promoting the holistic development of the program participants and different stakeholders have positive perceptions of the program [18–20]. 

The two courses described in Shek [3] have been approved to be General Education courses by The Hong Kong Polytechnic University. The first course on leadership is piloted in 2010–2012 academic years, and systematic teaching materials on self-understanding and interpersonal communication will be developed. The objectives of the course include: (1) to enable students to learn and integrate theories, research and concepts of the basic personal qualities (particularly intrapersonal and interpersonal qualities) of effective leaders; (2) to train students to develop and reflect on their intrapersonal and interpersonal qualities; and (3) to promote the development of an active pursuit of knowledge on personal qualities in leadership amongst students. On successfully completing this subject, students will be able to: (1) understand and integrate theories, research, and concepts on the basic qualities (particularly intrapersonal and interpersonal qualities) of effective leaders in the Chinese context; (2) develop self-awareness and understanding of oneself; (3) acquire interpersonal skills; (4) develop self-reflection skills in their learning; and (5) recognize the importance of active pursuit of knowledge on intrapersonal and interpersonal leadership qualities. 

The positive youth development constructs covered in the course included self-understanding, emotional competence, cognitive competence, resilience, spirituality, social competence, moral competence, positive identity, interpersonal communication, conflict resolution, relationship building, and assertiveness. Through lectures, class activities, and assignments, students are helped to understand the attributes of a successful leader, conduct personal reflections, and cultivate their awareness of the importance of intrapersonal and interpersonal attributes of a successful leader. As the developmental level of university students is very different from that of junior secondary school students, the teaching materials are fundamentally different from those used in the P.A.T.H.S. Project in Hong Kong. In other words, although the positive youth development constructs used in the Project P.A.T.H.S. and the present course are highly similar, the content is fundamentally different because of developmental differences in the two populations.

## 4. Implementation and Evaluation of the Pilot Course

The proposed subject has been piloted in the second term of 2010/11 school year. The subject was offered to four classes of students, with a total of 268 students (65 in Class A, 68 in Class B, 66 in Class C, and 69 in Class D).

Evaluation plays an indispensable part in assessing the value of this course. Adopting the criteria based on positivism and postpositivism, Biglan et al. [21] suggested that there are different levels of evidence that researchers and practitioners can consider. Grade 1 evidence is met when the preventive intervention is implemented in its intended setting with sufficient staff training and monitoring of implementation and outcomes. For Grade 2 evidence, two or more independent research teams are involved in multiple well-designed, randomized, controlled trials or multiple well-designed, interrupted time-series experiments. For Grade 3 evidence, a single research team is involved in multiple well-designed, randomized, controlled trials or multiple well-designed, interrupted time-series experiments. For Grade 4 evidence, it refers to at least one well-designed, randomized, controlled trial or an interrupted time-series design that is replicated across three cases. For Grade 5 evidence, it refers to evidence based on nonequivalent group design (i.e., participants are not randomly assigned to the experimental group and control group). For Grade 6 evidence, it refers to the use of preexperimental design without the involvement of a control group. Finally, Grade 7 evidence refers to clinical experience by respected researchers and practitioners and case reports.

Ideally, randomized groups trials in multiple sites conducted by different teams of independent researchers should be attempted. However, there are several practical difficulties involved. First, it is very expensive to conduct randomized group trials in different settings. Second, it is difficult to assign schools and clients to the control group (e.g., some schools may refuse to join the control group). For example, McCall et al. [22] reviewed three major summative approaches to evaluation (randomized experimental designs, nonequivalent control group designs, and interrupted time-series designs) and remarked that “methodologically, it is now acknowledged that conducting robust true experiments in the field—the scientific ideal described at the beginning of this chapter—is extremely difficult and often impossible” (p. 982) and “in response to these difficulties, alternatives to the true randomized experiment, especially utilization of the “quasi-experiment” (i.e., investigations not involving random assignment of participants), have become extremely widespread” (p. 983). Third, randomized controlled trials may not be the best strategy for positive youth development programs at the earlier stage of a program. Finally, for researchers not upholding the tenets of positivism or postpositivism, randomized control trial may not be regarded as a superior form of strategy that can yield knowledge about the program effect. For example, there are increasing voices criticizing the sole reliance on experimental methods and arguing for the use of more diverse types of evaluation strategies, particularly in the context of education [23].

Because postpositivistic evaluation approach is the dominant approach in the field, a postpositivistic paradigm (i.e., critical realist paradigm) with the use of multiple evaluation strategies will be adopted. There are several features in postpositivism. Ontologically speaking, critical realism (i.e., “real” reality but only imperfectly and probabilistically apprehensible) is adopted. Epistemologically speaking, a modified dualist/objectivist standpoint, with emphases on critical tradition and community, is highlighted. Methodologically speaking, critical multiplism (i.e., different methods including qualitative methods are used) is upheld. Consistent with the spirit of triangulation, different evaluation strategies were used to evaluate the pilot course:

**(i) Objective Outcome Evaluation (One-Group Pretest-Posttest Design)**

Pretest and posttest data utilizing a one-group pretest-posttest design were collected from the students taking the course. Students completed the questionnaires in a voluntary manner with informed consent. Thirteen subscales in the Chinese Positive Youth Development Scale were used as outcome measures. These include resilience, social competence, emotional competence, cognitive competence, behavioral competence, moral competence, self-determination, self-efficacy, beliefs in the future, clear and positive identity, spirituality, bonding, and prosocial norms. With reference to the findings based on multigroup confirmatory factor analyses [24], these subscales could be subsumed under three composite measures based on higher-order factors, including cognitive-behavioral competencies (CBC), positive identity (PI), and general positive youth development qualities (GPYDQ). A total of 50 successfully matched questionnaires were collected.

**(ii) Postlecture Subjective Outcome Evaluation**

At the end of each lecture, students were invited to respond to a subjective outcome evaluation form on their perceptions of the content of the lecture and their views. There are 12 items and one open-ended question in the evaluation form. The items cover assessment in the areas of lecture design, atmosphere, peer interaction, interest in content, student participation, opportunities for reflection, degree of helpfulness to personal development, instructor's mastery of lecture, varied teaching method, knowledge helpful to students, global evaluation of lecture, and global evaluation of the lecturer. A total of 2,039 questionnaires were collected for all lectures throughout the course.

**(iii) Postcourse Subjective Outcome Evaluation**

At the end of the course, students were invited to respond to a subjective outcome evaluation form including items assessing their perceptions of the course (10 items), instructor (10 items) and perceived effectiveness of the program (17 items). The form has been validated in other positive youth development programs in Hong Kong [17]. A total of 189 questionnaires were collected.

**(iv) Process Evaluation**

In process evaluation, systematic observations were carried out by two trained colleagues to understand the program implementation details in 14 lectures. In particular, adherence to the curriculum was examined. The 13-item Curriculum Delivery Assessment Scale was used to measure the quality of program delivery in the areas of student interest, student participation and involvement, classroom control, use of interactive delivery method, use of strategies to enhance student motivation, use of positive and supportive feedback, instructors' familiarity with the students, opportunity for reflection, degree of achievement of the objectives, time management, quality of preparation, overall implementation quality, and success of implementation with a 7-point scale for each item. The evaluation form has been used in similar positive youth evaluation studies in Hong Kong [25].

**(v) Qualitative Evaluation (Focus Groups Based on Students)**

Focus groups involving students based on schools randomly selected from the participating schools were carried out. A total of five focus groups based on different groups of students (*N* = 8, 8, 2, 3, and  2, resp.) have been conducted.

**(vi) Qualitative Evaluation (Reflection Notes)**

At the end of the subject, students were invited to use three words or phrases to describe their feelings, perceptions, and experiences of this course. Furthermore, they were asked to think about a metaphor (i.e., some object, event or state) which could stand for the course (e.g., an enjoyable tour, buffet, compass in life etc.) and gave a brief explanation about the meaning of the metaphor. A total of 189 pieces of reflection notes were collected.

## 5. Initial Evaluation Findings

Quantitative evaluation findings based on objective outcome evaluation, postcourse subjective outcome evaluation, and process evaluation are presented in this paper. It is noteworthy that the paper represents a brief overview of the different areas of evaluation. Readers can see the detailed findings in the papers generated from the project.

**(i) Objective Outcome Evaluation**

Shek and Sun [26] showed that the participants showed increase in scores in the total scale, subscales, and several other composite scores based on the Chinese Positive Youth Development Scale. Using composite variables (CBC, PI, and GPYDQ) derived from confirmatory factor analyses as indicators, analysis using MANOVA showed that the mean posttest score was higher than mean pretest score (Omnibus *F* = 11.79, *p* < .01). Further analyses using one-way ANOVA showed that posttest score was higher than pretest score in CBC (*F* = 8.36, *p* < .01; 4.57 and 4.78 for mean pretest score and mean posttest score), PI (*F* = 7.82, *p* < .01; 4.60 and 4.80 for mean pretest score and mean posttest score), and GPYDQ (*F* = 9.55, *p* < .01; 4.80 and 4.97 for mean pretest score and mean posttest score).

**(ii) Postcourse Subjective Outcome Evaluation**

The quantitative findings are presented in Shek and Sun [27]. Reliability analyses showed that the items assessing the program (10 items), instructor (10 items) and effectiveness (17 items) were internally consistent (alpha = 0.97; mean interitem correlation = 0.45). The findings also showed that the students had positive evaluation of the content of the course and the instructors. Furthermore, students generally perceived the course to be beneficial to their development. For example, roughly 90% of the respondents had positive evaluation of the course; roughly 98% had positive evaluation of the instructor; 94% of the students perceived the course as enriching their lives. Using the composite scores derived from the perceived program attributes (10 items), perceived worker attributes (10 items) and perceived program effectiveness (17 items), Pearson correlation showed that these three domains were inter-related (*r* = .56, *p* < .0001 for the relationship between perceived course attributes and perceived instructor attributes; *r* = .73, *p* < .0001 for the relationship between perceived course attributes and perceived effectiveness; *r* = .45, *p* < .0001 for the relationship between perceived instructor attributes and perceived effectiveness). Multiple regression analyses further showed that while course predicted perceived effectiveness (beta = .70, *p* < .0001), perceived qualities of the teacher did not (beta = .05, nonsignificant).

**(iii) Process Evaluation**

Shek and Sun [28] reported findings based on process evaluation of the study and several observations can be highlighted from the findings. First, the 13-item process evaluation scale was found to be internally consistent (alpha = 0.94, mean inter-item correlation = 0.55). Second, consistency of the two raters on program adherence was on the moderate range (Spearman *r* = 0.56, *p* < 0.05). Third, the mean ratings for different lectures were generally positive (*M* = 5.25 for Student Interest, *M* = 5.39 for Student Participation and Involvement; *M* = 5.43 for Classroom Management; *M* = 5.50 for Interactive Delivery Method; *M* = 5.51 for Strategies to Enhance Student Motivation; *M* = 5.18 for Use of Positive and Supportive Feedback; *M* = 4.43 for Familiarity with the Students; *M* = 5.50 for Opportunities for Reflection; *M* = 5.25 for Achievement of Lecture Objectives;  *M* = 5.11 for Time Management; *M* = 5.61 for Lecture Preparation; *M* = 5.39 for Overall Implementation Quality; *M* = 5.21 for Success of Implementation). Third, the rates for program adherence were quite respectable. The mean adherence rate across lectures was 86%.

**(iv) Qualitative Evaluation (Reflection Notes)**

Shek and Sun [29] showed that the participants had positive perceptions of the course and they used positive metaphors to describe the course.

## 6. Discussion

The present paper outlines the background and the content of the course entitled Tomorrow's Leaders offered at The Hong Kong Polytechnic University with reference to the issue of holistic development in university students in Hong Kong. Besides, different evaluation mechanisms are also outlined. There are several unique characteristics of this course. First, it is a pioneering and innovative course as there are few credit-bearing courses focusing on intrapersonal competencies in university students. Second, four classes of students were involved in the program evaluation. Third, several evaluation mechanisms were used to examine the effectiveness of the course. Finally, the findings are generally positive, suggesting that the students perceived the course to be beneficial to their own development and there was improvement in indicators of positive youth development.

 For the objective outcome evaluation, findings showed that the mean posttest scores based on the composite measures derived from confirmatory factor analyses were higher than those at pretest. That is, there were positive changes in cognitive-behavioral competencies, positive identity, and general positive youth development qualities in the program participants. Of course, as it is a study based on preexperimental design, it is noteworthy that there are alternative explanations (e.g., natural maturation) involved. Nevertheless, the findings can be regarded as encouraging at the beginning phase of the project.

For the postcourse subjective outcome evaluation, results showed that the respondents had positive views about the course, instructors, and perceived effectiveness [27]. Nevertheless, there are three alternative explanations for the present positive outcomes. First, students might be frightened of punishment if they voiced any unfavorable views. Second, students might act in a cooperative manner (i.e., demand characteristics). Third, students might not respond in a serious manner, which contribute to the high proportion of positive responses. However, all these alternative explanations could be dismissed for several reasons. First, student participation was voluntary and anonymous and there is no personal identifier in the questionnaires. Second, negative ratings were in fact recorded, as revealed from the quantitative and qualitative data. Finally, reliability analyses showed that the whole scale was internally consistent.

Consistent with previous studies, both program factors were significantly related to the perceived program effectiveness based on Pearson correlation analyses. These findings supported the notion that effective program implementation is multidimensional [30]. However, relative to program implementers, program quality was a stronger predictor of perceived effectiveness and the amount of variance that program implementers could explain perceived program effectiveness was small. This tentatively suggests that program content appeared to be more influential in affecting program effectiveness as compared to the perceived qualities of program implementers. Durlak and DuPre [30] argued that most of the intervention studies failed to examine the relative influence of different factors associated with program effectiveness. The results of the present study are a positive response and attempt to fill this research gap. As there are few studies on the predictors of perceived effectiveness of positive youth development programs, the present study can be regarded as pioneer in nature.

For the process evaluation findings, consistent with previous studies, the present study showed that the 13-item measure was internally consistent. Besides, different aspects of the program were perceived to be very positive. These aspects included students' interest and involvement (item 1 and item 2), management and teaching strategies used by the instructors (items 3 and 4), reflection (item 8), and lecture preparation (item, 11). In addition, the observers perceived that the objectives of the units implemented could be achieved (item 9), the overall quality of implementation was high (item 12), and the implementation was successful (item 13). However, there are several items over which the mean ratings were comparatively lower (items 5, 6, 7, and 10). In particular, familiarity with the students and time management were issues that could be improved. Finally, results showed that the overall degree of adherence to the teaching units assessed by the two observers was high. This point is important because program adherence is always a big problem in positive youth development programs in Western contexts.

Of course, we do not argue that the course on self-understanding and interpersonal development alone is adequate to promote positive development in university students. Actually, there are research findings showing that other courses such as service learning can help university students to develop mature behavior and civic responsibilities. However, our argument is that in carrying out service learning tasks, students should have some basic qualities such as self-understanding and interpersonal communication skills in the first place. Therefore, one should realize the intimate link between positive youth development and other student development programs such as service learning.

Another issue concerns whether the course should be credit-bearing. There are two arguments supporting this arrangement. First, as research finding showed that university students show many problems such as egocentrism and lack of civic responsibilities, offering a credit-bearing course can systematically respond to these issues. This strategy is consistent with the spirit that “no child should be left behind”. Second, if there is no credit attached to the course, students will simply lack motivation to study and treat the course in a non-serious manner.

It is noteworthy that there are some limitations of the study. First, although the sample size was not small, only four classes of students were involved in this study. Therefore, one should be cautious about generalizability of the findings. Second, the restricted response format of the closed-ended questions would limit the respondents' expression of negative experiences, and the quantitative findings would hardly unravel the inner world of the respondents. Hence, in order to illuminate the quantitative findings, further effort to examine such negative responses by looking at the qualitative findings is necessary. Third, if resources permit, addition of a control group can further clarify the impact of the program on the development of the program participants. Despite these limitations, the present findings fill up the research gap of the perceived effectiveness of a positive youth development course. In response to the comment of Chickering [31] that colleges and universities “have generally ignored outcomes related to moral and ethical development as well as other dimensions of personal development” (p. 1) and “have failed to graduate citizens who can function at the levels of cognitive and moral, intellectual, and ethical development that our complex national and global problems require” (p. 3), this credit-bearing course is a timely response. It is our humble wish that our experience in Hong Kong can serve as a modest example for other colleagues who are passionate about holistic development in university students.
